# Zonnic®: a new player in an old field

**DOI:** 10.1186/s13011-017-0124-3

**Published:** 2017-09-06

**Authors:** Min Gong, Michael S. Dunbar, Claude Setodji, William G. Shadel

**Affiliations:** RAND Corporation, 4570 5th Avenue, Suite 600, Pittsburgh, PA 15213 USA

**Keywords:** Tobacco control, Tobacco industry, Nicotine replacement therapy product

## Abstract

The tobacco industry is continually evolving to adapt to increasing tobacco control pressure and regulation, and to cater to consumer preferences. Recently, RJ Reynolds rolled-out a nicotine-containing gum, Zonnic®, which is marketed as a smoking cessation and reduction product and is sold at convenience stores at a lower price and in a smaller quantity than existing brands sold at pharmacies. The introduction of Zonnic® products is a critical first step in tobacco industry’s involvement in the NRT market and a serious indication of the evolving tobacco and nicotine-delivery industry and environment. It is likely that this trend will continue and spread, and as such, have a significant impact at multiple dimensions, including consumer perceptions and behavior, tobacco and NRT industry business strategy, and regulation and policy. In this special communication, we present an overview of the current marketing strategy for Zonnic®, discuss its potential impacts at the market level and at the level of the individual consumer, and suggest research and policy priorities based on the magnitude and urgency of the impacts.

## What this paper adds

- Provides a detailed overview of Zonnic®‘s current marketing mix along four standard dimensions: product, price, place, and promotion.
- Argues that Zonnic® is likely an integral part of a much wider innovation movement at the tobacco industry.
- Describes how Zonnic® could impose significant impacts at the industry level and on individual consumers.
- Provides a blueprint for empirical research in this newly emergent domain of inquiry.

## Background

The tobacco industry has a long history of successfully adapting to pressure from the tobacco control community, and catering to -as well as shaping- the evolving preferences of the consumer [1–3]. Once strictly focused on combustible cigarettes and smokeless tobacco products like moist snuff and chewing tobacco, the industry is aggressively developing reduced-harm and smoking-cessation products and devices. These activities go beyond the pursuit of “light” and “ultralight” cigarettes (which were not safer than so-called “regular” cigarettes) and chewing tobacco and moist snuff to refining and developing technologies that allow individuals access to noncombustible forms of nicotine [4]. The major tobacco companies have an explicitly stated business goal of developing harm-reduction products that involve electronic cigarettes, inhaled nicotine, dissolvable nicotine products, and even nicotine replacement.

Recently, the tobacco industry has stepped into the nicotine replacement therapy (NRT) market. In 2009, Reynolds American Inc., the second largest tobacco company in the United States and the makers of Camel and Newport cigarettes, among others, bought Niconovum AB, a Sweden-based company making NRT products, for $44.5 million. In September 2012, Reynolds started test marketing its first nicotine replacement therapy (NRT) product, Zonnic® nicotine gum, in retail outlets at Des Moines, Iowa. Zonnic® gum was marketed as a smoking cessation *and* reduction product and came in three flavors (mint, fruit, and cinnamon) and two doses (2 mg and 4 mg). In 2014, Zonnic® was rolled out nationally, fulfilling Reynolds’ original market plan to go national with distribution [5]. This marks Reynolds’ official entry into the U.S. NRT market.

The CEO of RJ Reynolds was quoted as saying that Zonnic® represents a step toward the company becoming a “total tobacco company.” In 2015, Reynolds formed a partnership with Pinney Associates, a healthcare consulting firm with a large research portfolio in tobacco control, and affiliate JSR LLC, a pharmaceutical product development company, to work on “products, regulations and policies related to smoking cessation and harm minimization … for transforming the tobacco industry” [6]. Recently, Reynolds rolled out their second NRT product, Zonnic® nicotine mini lozenge. As such, Zonnic® is likely the first of several reduced-harm products developed and mass-marketed by the major tobacco companies.

A recent paper by Kostygina and colleagues calls for attention to the difference between Zonnic® and traditional NRT, raises concerns that Zonnic® marketing may “blur the line” between NRT and smokeless tobacco products, and identifies relevant regulation issues [7]. In this review, we extend the previous research by approaching the problem from a systematic marketing perspective and providing an in-depth overview of the current marketing strategy for Zonnic® in the standard four-P (Product, Price, Place, and Promotion) marketing mix framework [8]. We also discuss Zonnic®‘s potential impacts at the market level (both the NRT market and tobacco market) and at the level of the individual consumer, and suggest a blueprint for research and policy priorities in this newly emergent domain of inquiry. We view this review, with a focus on Zonnic® marketing strategy and its impacts on consumer perception and relevant industries, and the Kostygina paper, which focuses on regulation, as complimentary work, and hope the current research, together with the Kostygina article, will prompt more discussion and research on this new trend, and equip the tobacco control community with better scientific knowledge for future regulation, should the need arise.

## ZONNIC®’s unique market positioning and strategy

Zonnic® was a milestone move in the tobacco industry’s involvement in the NRT market and its business development plan. It has a unique mix of the four P’s [8] as its marketing strategy: product, price, place, and promotion. At the *product* dimension, Zonnic® products are essentially the same as the NRT products that have been available in the U.S. for decades. Similar to other NRT makers (e.g. GlaxoSmithKline), Reynolds has paid much attention to Zonnic® product details to signal its quality, such as including a detailed, easy to navigate instruction sheet in the package and offering three popular flavors and two dosages for the gums. However, Zonnic® possesses two unique product features, compared to the traditional NRT products. First, the maker is a major tobacco company instead of a pharmaceutical company, the traditional NRT producer. Zonnic® is the first NRT product manufactured and marketed by a major tobacco company with Food and Drug Administration (FDA) approval. Under a new brand (Zonnic®), it is a product development strategy Reynolds has applied to diversify its product portfolio and explore new markets. Second, Zonnic® was designed to be sold in smaller packages. Currently both Zonnic® gum and mini lozenge are sold at 10 pieces, while most of other nicotine gums and nicotine lozenges are sold at 20 pieces or more.

Consistent with smaller packages, Zonnic® products are sold at lower *prices* than traditional NRT products. The manufacturer suggested retail price (MSRP) is $4.99 for Zonnic® gum and $5.49 for Zonnic® mini lozenge. Many retailers sell the products at a significantly lower price than the MSRP. For example, at the time of writing this paper, Zonnic® gum and mini lozenge are sold at $4.48 and $4.98 at Walmart in Pennsylvania. RiteAid.com, the internet shopping portal for one of the biggest drug store chains in the U.S., has Zonnic® gum for sale at $3.59. By comparison, most other nicotine gums are priced between $20 and $40 for 100 pieces, and nicotine lozenge $10 or more for 20 pieces. Note that the unit price of Zonnic® (without point of sale (POS) promotion discounts, such as coupons) is comparable to other NRT products – the lower retail prices result from smaller packages rather than lower unit price.

**Fig. 1:**
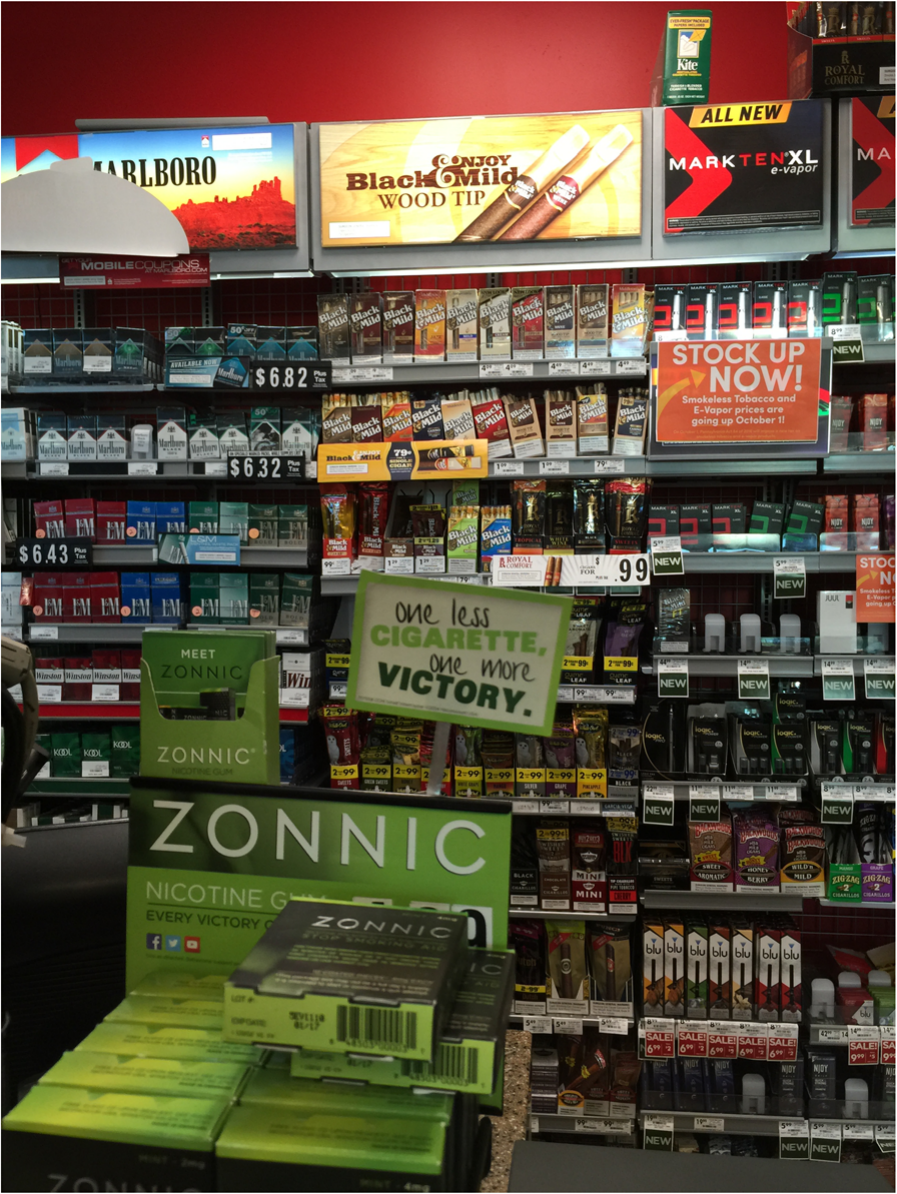
Zonnic® in a convenience store in Pennsylvania

Zonnic® also has a unique distribution channel combination (*place*) compared to traditional NRT products. Originally, Zonnic® was designed to be sold in convenience stores and gas stations, but quickly became available at some other retail locations. For example, in Pennsylvania, Zonnic® is either displayed in the pharmacy section, separated from the tobacco sales lane (e.g. in grocery stores), or sold alongside traditional tobacco products (e.g. in convenience stores, gas stations, Walmart stores, and pharmacy stores). Zonnic® is now specifically marketed as being available “wherever cigarettes are sold,” and has 33,000 retail outlets across the United States [9]. The presence of Zonnic® at convenience stores and gas stations are of particular interest, as traditional NRT products are usually not sold in those retail locations. Moreover, at convenience stores, Zonnic® is usually displayed close to the tobacco power wall, but in a much more prominent fashion (Fig. 1). Unlike Nicorette and other traditional NRT products, Zonnic® is currently *not* widely sold on the internet, although it is available through some specific retailer web portals (e.g. RiteAid.com).

In terms of *promotion*, Zonnic® has been following many common practices of marketing, including offering free samples on industry websites, providing dollar-off coupons at exhibitions, and banner advertising at POS (Figure 1). A combination of pushing and pulling strategies [10] have been applied to take advantage of the existing distribution channel to present Zonnic® physically in front of consumers, use media advertising to increase consumer awareness of the product, and utilize search engines and social media platforms to attract consumers. Of particular interest is Zonnic®‘s choice of its advertising theme. Zonnic® commercials use the message “Take it one less cigarette at a time”, which is consistent with a gradual cessation approach. The product’s website specifically encourages long-term use and simultaneous use with other nicotine products, with references to the FDA’s own documents on the safety of long-term use of nicotine replacement while reducing cigarette consumption [11]. By comparison, traditional NRT product instructions are geared toward total replacement, rather than substitution of individual cigarettes (i.e., dual use for purposes of cigarette reduction as opposed to cigarette cessation).

To summarize, Zonnic® has created a unique marketing mix as it enters the NRT market. Although there is no data available yet on Zonnic® sales, in the most recent RAI report to investors, its president and chief executive officer said: “the national expansion of Zonnic®’s nicotine replacement therapy gum is (also) progressing smoothly.” [12].

## Potential impact of Zonnic®

The introduction of Zonnic® into the NRT market may have an impact at multiple levels, including consumer perceptions and behavior, tobacco and NRT industry business strategies, and regulatory implications.

### How might Zonnic® influence consumer perceptions and purchase of NRT?

Zonnic® may have mixed effect on consumers’ decisions to use NRT. The presence of Zonnic® products and promotion at POS locations (i.e. convenience stores) may serve as a cue to quit smoking and increase NRT use. Moreover, smaller packages and lower price of Zonnic® may increase the accessibility and affordability of NRT products, especially for those who had difficulty affording a larger package at a time, and those who were not aware of the role of NRT products in cessation. This may incentivize purchasing among lower-income adult smokers (e.g., older adults on a fixed income who want to quit smoking). Zonnic® may also influence NRT products at the market level. Its entrance contributes to the creation of a more competitive NRT market for pharmaceutical companies, which may further improve the NRT accessibility to the general population.

However, increasing accessibility could have unintentional consequences for public health. For example, there might be increased use of NRT products for non-cessation purposes, such as recreational use, weight loss, or situational use to circumvent smoking restriction and ban. In addition, lower pricing and smaller packages may incentive use among price-sensitive youth. Further, the novelty of these products in certain retail environments (e.g., convenience stores) may appeal to non-tobacco users, potentially increasing the pool of nicotine consumers. Moreover, an NRT product produced and marketed by a tobacco company may be viewed as a substitute for smoking, rather than as a replacement. Consumers may begin to align NRT products not with quitting smoking, but as another type of tobacco product that can substitute for smoking (e.g., akin to e-cigarettes), or products to be used in conjunction with smoking to reduce overall risk. This can cause a conceptual shift for the public in terms of how NRT is traditionally viewed (i.e., as a cessation aid). This change in perception could favor reduction and dual use over cessation, which could lead to more/longer term NRT use, delayed cessation, prolonged nicotine dependence, etc.

For attempting quitters, the introduction of Zonnic® may impact their perception of the effectiveness of the NRT products, since Zonnic®, if effective, could cannibalize Reynolds primary tobacco products, which might be seen to be against Reynolds’ long-term business interests. This apparent conflict of interest could lead consumers to doubt the efficacy of NRT products for reducing cigarette use, which could prevent smokers from resorting to Zonnic® or perhaps other NRT products for quitting, despite FDA approval of NRT products as aids to smoking cessation and considerable scientific evidence supporting the efficacy of NRT [13]. In addition, many smokers have aversions to using NRT and have a strong desire to discontinue nicotine entirely, in part due to the belief that switching from cigarettes to NRT will not end their addiction to nicotine [14]. Smokers also hold erroneous beliefs about the safety of NRT products (e.g., that nicotine is the primary harmful substance in tobacco [14, 15]). Portraying NRT as an alternative product from a tobacco company could reinforce the belief that NRT is just another type of nicotine or tobacco product, which could influence beliefs about the efficacy and safety of NRT and dissuade some smokers from using it as a cessation aid.

### How could Zonnic® influence smokers’ choice of cessation approach?

There is conflicting evidence as to whether a gradual or abrupt approach to quitting smoking is more effective. For example, a Cochrane review found equal cessation rates among the two methods, independent of NRT use or behavioral support [16]. However, more recently, Lindson-Hawley and colleagues report that the gradual cessation approach is not as effective as the abrupt approach, in both the short and longer term. Gradual cessation may deter smokers from making quit attempts [17].

It is conceivable that Zonnic®‘s central marketing theme of “Take it one less cigarette at a time” could influence consumers’ decision about whether to choose a gradual or abrupt approach for quitting. The “small steps” advertising may also change consumers’ perception on the definition of cessation [7]. Will cutting down and co-use of combustible tobacco and NRT lead to complete abstinence or an acceptable alternative to cessation? Although described as “stop smoking aids” on the product website (www.zonnic.com), note that none of the Zonnic®‘s commercials or user guides link Zonnic® use to cessation counseling or behavioral support, which, when added to NRT, can facilitate the likelihood of quitting [18]. However, Zonnic®‘s website *does* link to FDA statements regarding the efficacy of NRT and guidelines for use, and includes the statements that “if after 12 weeks you feel you need to keep on with Zonnic®® a little bit longer, you can. Just be sure you first consult with your healthcare professional” and “Behavioral support program increases chances of success.”

### How could Zonnic® influence POS tobacco marketing?

In 2012, the tobacco industry spent nearly $8 billion at POS, and nearly $500 million was devoted to ensuring products are prominently placed in the retail environment on tobacco power walls [19, 20]. Previous research has shown that locating tobacco power walls conspicuously behind the cash register increases the likelihood of consumers being repeatedly exposed to positive tobacco messages [21]. These exposures are thought to normalize tobacco use [4], increase brand recognition [21], and increase positive brand user imagery [22]. Exposure to tobacco power walls can have a potent effect on teens’ thoughts and feelings about smoking [23–26].

Theoretically, the presence of Zonnic® close to the tobacco power wall could disrupt the salience of pro-tobacco products by interfering with cognitive processing of the pro-tobacco “message” conveyed by the power wall (e.g., that tobacco use is normative): The “clutter” produced by competing messages leads to less certainty about the overall message in a particular domain and, thus, negatively influences norms and attitudes and purchase intentions for the product in question [24]. It follows, then, that the more closely linked the competing messages, the greater the uncertainty and more negative the tobacco use cognitions [27]. Hence, Zonnic® might serve as a reminder of the addictive nature of cigarettes, convey an anti-tobacco cue (that it is desirable to quit), and reduce the influence of the tobacco power wall. This potential anti-tobacco benefit could be significant, considering the fact that Zonnic® is the first NRT brand to be massively marketed and prominently displayed close to the tobacco power wall at convenience stores, and nearly 50% of all tobacco products are sold at convenience stores. [28]

### How could Zonnic® influence consumer perception of Reynolds and tobacco industry?

Zonnic® may change consumer perceptions of its makers, Reynolds, and improve the public image of tobacco companies as being more socially responsible. Such an effect would mirror the public relations campaign the tobacco industry waged in the 1990s with a focus on social responsibility, support for restrictions on youth access, and development of smoking prevention programs [29]. There is evidence such efforts did little to diminish youth smoking [30] but did improve the public’s perceptions of smoking and the tobacco industry [31]. Zonnic® and Reynolds cigarette brands may form a positive feedback loop on consumer perceptions. On the one hand, Zonnic® may earn the confidence of smokers, because it comes from the same manufacturer as some of their favorite brands, which they trust already. On the other hand, smokers may develop more positive attitudes toward Reynolds cigarette brands, because the company produces harm reduction products, an indication of care for customer health and product quality. Such positive views may improve customer loyalty to Reynolds cigarette brands.

Interestingly, Reynolds is currently taking a neutral stand on its ownership of Zonnic®; it does not hide it nor publicize it. None of the Zonnic® commercials mention Reynolds. However, there were multiple news stories and interviews (with Reynolds personnel) that highlighted Reynolds as the maker of Zonnic®. The progress on Zonnic® was repeatedly mentioned in RAI’s public earning reports to its investors.

Table 1 summarizes some potential impacts discussed above at both market and individual levels. Note that we do not intend to provide an exhaustive list, or conclusions based on actual data points. Some hypothesized impacts are on the longterm dimension and cannot be confirmed or refuted in the near future. Others need innovative research and data collection to test the hypotheses scientifically.

**Table 1:** Hypothesized impact of Zonnic®

Market Level	NRT Industry	• More competitive NRT market • Diversified NRT products • Increased NRT availability • New users, both for cessation and non-cessation purposes
	Tobacco Industry	• Loss of cigarette customers • Increase in dual cigarette-and-NRT consumers • Product diversification for better risk management • Improved public image of social responsibility
Individual Consumer	Non-smokers	• Reduced impact from tobacco powerwall marketing (higher awareness of the addictive nature of smoking, lower perception of smoking prevalence and social approval, etc.) • Increased use of NRT for non-cessation purposes (recreational, weight loss)
	Smokers	• Higher perceived smoking harms • Higher perceived ease of quitting smoking • Increased likelihood for quit attempt • Increased preference for gradual reduction approach • Changed perception on what counts as “cessation” • Higher awareness and use of NRT products for cessation • Increased dual use of NRT and cigarettes (e.g., for circumventing smoking restrictions) • Higher brand loyalty to Reynolds and positive attitude toward tobacco industry

## Implications

### Regulation concerns on NRT and smokeless products

There are concerns that the new NRT products by tobacco companies blur the line between NRT and smokeless products [7]. For example, if a company markets a novel smokeless product as a modified risk tobacco product (MRTP), it will go through a MRTP review which requires significantly reduced risk for individual users and benefits for public health at the population level. Alternatively, the company can market the product as a cessation aid product, such as in the case of Zonnic®, which is subject to a different review process. Cessation aid products are reviewed by the FDA’s Center for Drug Evaluation and Research (CDER) for evidence that the product is safe and effective for its intended use; however, these products need not demonstrate that they “benefit the health of the population as a whole” [32]. Moreover, the CDER review can circumvent the MRTP regulation on pricing and sale distribution of tobacco products.

### Zonnic®’s role in Reynolds’ long-term business strategy

Facing long-term challenges and a changing environment in the tobacco industry, as well as a changing regulatory environment that may threaten the future profitability of combustible products, Reynolds has undergone substantial changes that impact its business development strategy. The company specifically adopts a “transforming tobacco” strategy, and has a dedicated website for this initiative, and claims that it will “lead change in our industry by driving innovation.” [33] Reynolds has taken significant steps in this direction, including its acquisition of Niconovum, introduction of American Snuff and VUSE, partnership with Pinney Associates, and more recently, the creation of a new subsidiary, RAI Innovations Co., which will focus on next generation vapor and nicotine products. Under the new structure, R.J. Reynolds Vapor Co. and Niconovum USA will be more integrated. That is, Zonnic® will be an integrated component of an initiative at the entire company level, and will be managed by the same subsidiary as Reynolds’ VUSE, which allows for centralized product development and improved marketing effectiveness to avoid common pitfalls such as sibling product rivalry and market cannibalization (e.g. between VUSE and Zonnic®). Note that VUSE is now the top selling vapor product in convenience store and gas stations, and available in more than 114,000 retail outlets across the nation. The success of VUSE was partly attributed to its “lower price (22% below the e-cigarette category average) thanks to heavy couponing efforts by Reynolds.” [34] It is possible that Zonnic® will tap into VUSE’s supply chain and distribution channels, and adopt similar marketing strategies, under the centralized management of the new subsidiary, RAI Innovations Co.

### On the credibility of tobacco Industry’s harm reduction talk (and walk)

Reynolds’ move on smokeless products and Zonnic® is an important part of its business development strategy to survive and prosper in a new era with a disappearing market base for its combustible products. Other major tobacco companies have taken similar steps. For example, Altria spoke publicly that “we have a strategy to grow new income streams with innovative products that meet those adult consumer preferences.”

It is worth noting that the core business of the tobacco industry remains on the combustible side. For example, in a presentation to investors, Reynolds stated “We spend about 80 percent of our resources in the combustible space. … despite a lot of these new innovations that you see coming out, 90 percent of our R&D budgets are actually directed at the combustible category…. That is the category that's still going to deliver a lot of growth into the future” [35].

Whether those innovative products are motived by adapting to seek a way out of a diminishing market, compensating for continued focus on combustible products, cultivating a more positive public image, increased interest in the public good, or a mix of motivations, the trend is likely to continue and we will see more innovative products enter existing markets or develop new markets. Given the lack of evidence on the long-term health impact of “innovative” products, it is difficult to weigh the benefits and costs from a public health perspective. In particular, it is unclear whether the innovation movement currently occurring in tobacco industry will help reduce population tobacco use and associated harms, or create “traps” to new generations of tobacco users.

## Conclusions

Although considerable attention has been paid to the impact of e-cigarette and other smokeless products [36], tobacco company marketed cessation products, e.g. Zonnic® gums and Zonnic® mini lozenge, have received little attention in the tobacco research literature. The potential impacts discussed in this paper are educated guesses, rather than scientifically supported facts. However, in the near future governments may face imminent decisions such as: should Zonnic® be allowed in state subsidized cessation programs? If Reynolds decided to publicize its involvement in the NRT market, should they be allowed to put their trademark on Zonnic® packages or even Zonnic® commercials? Those decisions can be better supported if we have the necessary information to evaluate the impacts in Table 1. Based on the potential impact on public health, we suggest that research priority should be given to the following questions:

- Assuming that the increased NRT accessibility encourages NRT use and cuts down combusted tobacco consumption, does such cutting-down behavior increase or decrease successful cessation (defined as complete removal of nicotine use)? What percentage of Zonnic® purchases are for non-cessation purposes?
- Does the presence and promotion of Zonnic® change smokers’ beliefs about the safety and efficacy of NRT, preferences for cessation approaches, and their perception of what counts as cessation? If so, at what magnitude?
- Are smokers, especially Reynolds customers, aware of the relationship between Zonnic® and Reynolds? If so, how does that change their perception of Reynolds and Reynolds brands?

In conclusion, Zonnic® is likely to be an integrated part of a bigger so-called “innovation movement” within the tobacco industry. Similar NRT and harm reduction products are anticipated in the future. Although Zonnic® can impose significant influences at multiple levels, little is known on the actual impacts. As such, it is important to monitor this trend and evaluate its effects on both individual consumers and at population level.

## Abbreviations

CDER: Center for drug evaluation and research
FDA: Food and Drug Administration
MRTP: Modified risk tobacco product
MSRP: Manufacturer suggested retail price
NRT: Nicotine replacement therapy
POS: Point of sale

## References

[1] Bialous SA, Peeters S (2012). A brief overview of the tobacco industry in the last 20 years. Tob Control.

[2] Cummings KM, Proctor RN (2014). The changing public image of smoking in the United States: 1964–2014. Cancer Epidemiol Biomarkers Prev.

[3] Gilmore AB. Understanding the vector in order to plan effective tobacco control policies: an analysis of contemporary tobacco industry materials. Tob Control. 2012;21(2):119–26. 10.1136/tobaccocontrol-2011-050397 PMC3705181 22345234

[4] McNeill A, Hammond D, Gartner C (2012). Whither tobacco product regulation?. Tob Control.

[5] CSPdailynews.com. Reynolds Readies Nicotine-Replacement Therapy Products. 2012. Lastly accessed on October 19^th^, 2016 at http://www.cspdailynews.com/category-news/tobacco/articles/reynolds-readies-nicotine-replacement-therapy-products.

[6] Winston Salem Journal. Reynolds American delving deeper into nicotine gum business. 2015. Lastly accessed on October 19^th^, 2016 at http://www.journalnow.com/business/reynolds-american-delving-deeper-into-nicotine-gum-business/article_8bce5918-b895-11e4-bb17-ffbdb76cb2d6.html.

[7] Kostygina G, England L, Ling P (2016). New product marketing blurs the line between nicotine replacement therapy and smokeless tobacco products. Am J Public Health.

[8] McCarthy EJ. Basic marketing: A managerial approach. Homewood, IL. RD Irwin; 1978.

[9] Reynolds American Inc. FORM 10-Q. For the quarterly period ended June 30, 2016. 2016. Lastly accessed on October 19^th^, 2016 at http://s2.q4cdn.com/129460998/files/doc_financials/2016/5e62c345-5e6a-4970-b5d8-fbf45ece0028.pdf

[10] Dowling GR (2004). The art and science of marketing: marketing for marketing managers.

[11] Zonnic®.com. What the FDA has to say. 2016. Lastly accessed on October 19^th^, 2016 at https://www.zonnic.com/Content/pdf/NRT_Label_Change.pdf

[12] Reynolds American Inc. Investor Relationship Correspondences. 2016. Lastly accessed on October 19^th^, 2016 at http://s2.q4cdn.com/129460998/files/doc_news/2016/2016-22-RAI-reports-strong-2Q16-perf-positive-outlook.pdf

[13] Moore D (2009). Effectiveness and safety of nicotine replacement therapy assisted reduction to stop smoking: systematic review and meta-analysis. BMJ.

[14] Vogt F, Hall S, Marteau TM (2008). Understanding why smokers do not want to use nicotine dependence medications to stop smoking: qualitative and quantitative studies. Nicotine Tob Res.

[15] Shiffman S (2008). Perceived safety and efficacy of nicotine replacement therapies among US smokers and ex-smokers: relationship with use and compliance. Addiction.

[16] Lindson N, Aveyard P, Hughes JR. Reduction versus abrupt cessation in smokers who want to quit. Cochrane Database Syst Rev. 2012;11:CD008033. 10.1002/14651858.CD008033.pub3 23152252

[17] Lindson-Hawley N (2016). Gradual versus abrupt smoking cessation. Ann Intern Med.

[18] Silagy C, et al. Nicotine replacement therapy for smoking cessation. Cochrane Database Syst Rev. 2004;3:CD000146. 10.1002/14651858.CD000146.pub2 15266423

[19] Federal Trade Commission (2015). Cigarette report, 2012.

[20] Dewhirst T (2004). POP goes the power wall? Taking aim at tobacco promotional strategies utilised at retail. Tob Control.

[21] Pollay RW (2007). More than meets the eye: on the importance of retail cigarette merchandising. Tob Control.

[22] Donovan RJ, Jancey J, Jones S (2002). Tobacco point of sale advertising increases positive brand user imagery. Tob Control.

[23] Henriksen L (2002). Effects on youth of exposure to retail tobacco advertising1. J Appl Soc Psychol.

[24] Kim AE (2013). Influence of tobacco displays and ads on youth: a virtual store experiment. Pediatrics.

[25] Shadel WG, et al. Hiding the tobacco power wall reduces cigarette smoking risk in adolescents: using an experimental convenience store to assess tobacco regulatory options at retail point-of-sale. Tob Control. 2016;25:679-84. 10.1136/tobaccocontrol-2015-052529 PMC4877296 26598502

[26] Wakefield M (2006). An experimental study of effects on schoolchildren of exposure to point-of-sale cigarette advertising and pack displays. Health Educ Res.

[27] Nan X, Faber RJ (2004). Advertising theory: Reconceptualizing the building blocks. Mark Theory.

[28] Center for Public Health Systems Science. (2014). Point-of-sale report to the nation: the tobacco retail and policy landscape. Center for Public Health Systems Science at the Brown School at Washington University in St. Louis and the National Cancer Institute, State and Community TOB CONTROL Research Initiative. Lastly accessed on October 19^th^, 2016 at: http://cphss.wustl.edu/Products/Documents/ASPiRE_2014_ReportToTheNation.pdf.

[29] Carter SM (2003). From legitimate consumers to public relations pawns: the tobacco industry and young Australians. Tob Control.

[30] Landman A, Ling PM, Glantz SA (2002). Tobacco industry youth smoking prevention programs: protecting the industry and hurting tobacco control. Am J Public Health.

[31] Farrelly MC (2002). Getting to the truth: evaluating national tobacco countermarketing campaigns. Am J Public Health.

[32] US Food and Drug Administration. Modified risk tobacco product applications–draft guidance for industry. Lastly accessed on October 19^th^, 2016 at: http://www.fda.gov/downloads/TobaccoProducts/Labeling/RulesRegulationsGuidance/UCM297751.pdf.

[33] Reynolds American Inc. (2016). RAI'S TRANSFORMING TOBACCO STRATEGY. Lastly accessed on October 19^th^, 2016 at: http://www.transformingtobacco.com/.

[34] CSPdailynews.com. (2014). Vuse Jumps to No. 1. Lastly accessed on October 19^th^, 2016 at: http://www.cspdailynews.com/category-news/tobacco/articles/vuse-jumps-no-1.

[35] seekingalpha.com. (2012). Reynolds American's CEO Hosts Investor Day (Transcript). Lastly accessed on October 19^th^, 2016 at: http://seekingalpha.com/article/1001691-reynolds-americans-ceo-hosts-investor-day-transcript.

[36] O’Loughlin J, Wellman RJ, Potvin L (2016). Whither the e-cigarette?. Int J Public Health.

